# Carbonic anhydrase-9 expression levels and prognosis in human breast cancer: association with treatment outcome

**DOI:** 10.1038/sj.bjc.6601122

**Published:** 2003-07-15

**Authors:** P N Span, J Bussink, P Manders, L V A M Beex, C G J Sweep

**Affiliations:** 1 Department of Chemical Endocrinology, University Medical Centre Nijmegen, Nijmegen, The Netherlands; 2 Department of Radiation Oncology, University Medical Centre Nijmegen, Nijmegen, The Netherlands; 3 Department of Medical Oncology, University Medical Centre Nijmegen, Nijmegen, The Netherlands

**Keywords:** breast neoplasms, quantitative real-time RT–PCR, hypoxia, survival analysis, prognosis

## Abstract

Here, we set out to assess *CA9* expression levels by real-time quantitative RT–PCR in breast cancer tissue samples obtained from 253 patients, and correlated those with relapse-free (RFS) survival. The median follow-up time was 75 months (range 2–168 months). *CA9* expression was mainly found in high-grade, steroid receptor negative cancer tissues. *CA9* levels were not significantly associated with RFS (*P*=0.926, hazard ratio (HR)=0.99, 95% CI=0.80–1.22) in the total cohort of 253 patients. In multivariate analysis with other clinicopathological factors, *CA9* (*P*=0.018, HR=0.77, 95% CI=0.62–0.96), the interaction of adjuvant chemotherapy with *CA9* (*P*=0.009, HR=1.31, 95% CI=1.07–1.61) and the interaction of adjuvant endocrine therapy with *CA9* (*P*<0.001, HR=1.41, 95% CI=1.20–1.66) all contributed significantly to the final model. These results indicate that patients with low *CA9* levels benefit more from adjuvant treatment than do patients with high levels. Thus, the determination of *CA9* levels could aid in the selection of patients who will not benefit from adjuvant therapy, and whose prognosis will more likely improve with other treatment modalities.

Hypoxia has long been known to play an important role in the development of treatment resistance in cancer. Hypoxic cells are particularly radioresistant (Höckel *et al*, 1996b), but hypoxia has also been associated with resistance to chemotherapy (Luk *et al*, 1990). Not only does tumour hypoxia directly attenuate therapy success, but it also leads to clonal selection of more aggressive cells leading to poor prognosis, independent of treatment modality (Höckel *et al*, 1996a; Rofstad, 2000). Molecular mechanisms underlying the association between hypoxia and treatment resistance are poorly understood. Identifying factors associated with treatment outcome in cancer patients provides the possibility to perform more effective therapies and offers targets for treatment resistance modification.

Carbonic anhydrase (CA)-9 is one of the best-known genes associated with tumour cell hypoxia, and is quickly and extensively upregulated under hypoxic conditions (Wykoff *et al*, 2000; Lal *et al*, 2001). CAs are a family of zinc metalloenzymes. The human gene product CA IX (MN or G250), originally identified in HeLa cells, catalyses the hydration of carbon dioxide to carbonic acid. CA IX is a transmembrane glycoprotein of 58/54 kDa, encoded by a 1.5 kb mRNA with exon 1 encoding a signal peptide and a proteoglycan-related region. Exons 2–8 code for a CA domain with a highly conserved active site. Exons 10 and 11 encode a transmembrane anchor and an intracytoplasmic tail, respectively (Opavsky *et al*, 1996). Studies have reported on CA IX expression in several carcinomas such as renal cell (Liao *et al*, 1997), colorectal (Saarnio *et al*, 1998), nonsmall lung (Vermylen *et al*, 1999; Giatromanolaki *et al*, 2001), cervical (Olive *et al*, 2001), bladder (Turner *et al*, 2002) and nasopharyngeal carcinoma (Hui *et al*, 2002), while being absent from most normal tissues. Its role in tumour growth and disease progression has been attributed to its effect on reducing pericellular pH in response to hypoxia, thereby facilitating the breakdown of extracellular matrix (Giatromanolaki *et al*, 2001), but CA IX might also play a role in cell–cell communications.

In ductal carcinoma *in situ* of the breast, CA IX can be detected (Wykoff *et al*, 2001). So far, only a few papers have addressed the expression of CA IX in invasive breast cancer (Chia *et al*, 2001; Bartosova *et al*, 2002). Here, we set out to assess *CA9* expression levels in a large number of samples by real-time quantitative RT–PCR, and correlated those with relapse-free survival (RFS) in patients who were treated with surgery alone, or with additional radio-, chemo- and/or endocrine therapy. Thereby, we aimed at identifying a potential predictive value of *CA9* for treatment success in breast cancer.

## MATERIAL AND METHODS

### Patients

Breast cancer tissue samples were obtained from 253 patients with unilateral, operable breast cancer who underwent resection of their primary tumour between February 1987 and December 1997. Selections were made based on availability of frozen tissue from eligible patients remaining in the tumour bank after routine oestrogen (ER) and progesterone receptor (PgR) ligand binding assay for five different hospitals of the Comprehensive Cancer Center East in the Netherlands. The median age was 60 years (range 31–88 years). Patients had no previous diagnosis of carcinoma (with the exception of basal cell carcinoma of the skin), no distant metastases at the time of diagnosis and no evidence of disease within 1 month after primary surgery. The latter patients are excluded because of the likely occurrence in this group of metastases at the time of primary surgery that escaped detection. Furthermore, patients receiving neo-adjuvant therapy or those with carcinoma *in situ* only were excluded. Patients underwent modified radical mastectomy (*n*=183) or breast-conserving therapy (*n*=70). Postoperative radiotherapy was given after mastectomy for T3-4 disease or in case of nodal involvement of ⩾3 nodes (*n*=114), and after BCT. Lymph node involvement was found in 131 patients. Subsequent systemic adjuvant therapy (85 endocrine therapy, 28 chemotherapy, 14 both) was given based on the consensus at the time of treatment. In the absence of involved axillary lymph nodes, patients usually did not receive adjuvant treatment. When involved axillary lymph nodes were detected, premenopausal patients received chemotherapy. In the case of an ER- and/or PgR-positive primary tumour, additional endocrine therapy was given to these patients. Postmenopausal patients with involved axillary lymph nodes and ER- and/or PgR-positive tumours received adjuvant endocrine therapy. If the primary tumour of these patients was hormone receptor negative, no adjuvant therapy was given, although in some cases endocrine therapy was opted for. The median follow-up time was 75 months (range 2–168 months). Patients were seen (history, physical examination, routine laboratory investigations) once every 3 months during the first 2 years, once every 6 months for the next 5 years and once a year afterwards. Once a year, X-ray mammography was made. During follow-up, 93 patients had a recurrence (20 locoregional, 71 distant metastases and two both) and 72 patients died of which 55 died due to breast cancer-related causes, two patients died by other malignancies, 15 patients died due to unknown causes. Contralateral breast cancer or second malignancies were not considered recurrent disease.

### Tissue processing

After primary surgery, a representative part of tumour was selected by a pathologist, frozen in liquid nitrogen and processed for routine ER and PgR ligand binding assay. These assays were performed as recommended by the European Organisation for Research and Treatment of Cancer (EORTC). Aliquots of tissue were kept in liquid nitrogen until RNA isolation.

### Immunohistochemistry

From the biopsy material, frozen sections of 5 *μ*m were cut and mounted in poly-L-lysine-coated slides and stored at −80°C until staining. Prior to staining, sections were fixed for 10 min in acetone of 4°C and rehydrated in phosphate-buffered saline (PBS). Between all consecutive incubation procedures, tissue sections were rinsed in PBS three times for 3 min. After rehydration, the sections were incubated with 5% normal donkey serum (Jackson Immuno Research Laboratories Inc., West Grove, PA, USA) in Monoclonal Liquid Diluent Immunostain (MLD, Euro/DPC Inc., Llanberis, UK) for 30 min at 37°C. Subsequently, they were incubated with mouse anti-human G250 (kindly provided by Dr Oosterwijk, Department of Urology, University Medical Center Nijmegen, The Netherlands) 1 : 50 for 45 min at 37°C. Next, the slides were incubated with donkey anti-mouse-alkaline phosphatase (alkaline phosphatase-conjugated affine pure donkey anti-mouse IgG (H+L), Jackson Immuno Research Laboratories Inc.) 1 : 100 in MLD for 60 min at room temperature followed by incubation with Vector red substrate (Vector Red Alkaline Phosphatase Substrate Kit I, Vector Laboratories, Burlingame, CA, USA) for 30 min at room temperature and by rinsing in tap water. Both avidin and biotin were blocked for 15 min at room temperature (Avidine/biotine blocking Kit, Vector Laboratories). For staining of the vessels in the same tissue, the sections were incubated in mouse anti-human CD34 (Coulter Immunotech, Marseille, France) 1 : 20 in MLD overnight at 4°C. After incubation with donkey anti-mouse-biotin (Jackson ImmunoResearch Laboratories Inc.) 1 : 500 in MLD, 60 min at room temperature, slides were incubated with the ABC reagent (Vector ABC-Elite kit standard, Vector Laboratories) and rinsed in deionised water for at least 5 min. The colour was developed with DAB (liquid DAB substrate kit, Zymed Laboratories, Inc S. San Francisco, CA, USA) for 5 min at room temperature. After rinsing in tap water, the sections were counterstained with haematoxylin (Klinipath, Duiven, The Netherlands) for 30 s. Finally, after rinsing in tap water again the tissue sections were dehydrated and mounted with KP mounting medium (Klinipath).

### RNA extraction

After pulverising by dismembration in liquid nitrogen, total RNA was isolated from approximately 20 mg tissue powder using the RNeasy mini kit (Qiagen, Hilden, Germany) with on-column DNase I treatment. The quality of the RNA was checked by examining ribosomal RNA bands after agarose gel electrophoresis, and by amplifying *β*-actin as a control (see below).

### Reverse transcription

Reverse transcription was performed using the Reverse Transcription System (Promega Benelux BV, Leiden, The Netherlands) according to the manufacturer's protocol. After annealing of random hexamers for 10 min at 20°C, cDNA synthesis was performed for 30 min at 42°C followed by an enzyme inactivation step for 5 min at 95°C.

### PCR

PCR reactions were carried out using Taqman Universal PCR master mix (PE Applied Biosystems, Nieuwerkerk a/d IJssel, the Netherlands) in a final volume of 25 *μ*l. In all, 50 nM of the *CA9* forward primer (5′-GAC GCC TGG CCG TGT TG-3′), and of the reverse primer (5′-CTG AGC CTT CCT CAG CGA TT-3′) and 200 nM of the Taqman probe (5′-TET-TTC TGG AGG AGG GCC CGG AAG A-3′) were used. Primers were from Biolegio BV (Malden, The Netherlands) and the Taqman probe from Perkin-Elmer (PE Applied Biosystems). The assay was designed intron-spanning using the Primer-Express software package version 1.5 (PE Applied Biosystems). *β*-Actin was amplified using the Pre-Developed Assay Reagents Taqman RT–PCR assay from Perkin-Elmer (PE Applied Biosystems). All amplifications, with denaturation at 95°C for 10 min, and 40 cycles of 15 s at 95°C (melting) and 60 s at 60°C (annealing and elongation), were performed on an ABI Prism 7700 Sequence detection system (PE Applied Biosystems). Quantification of unknown samples was performed using the colon cancer cell-line SW-480 as a calibrator.

### Statistical analyses

Statistical analyses were carried out using SPSS 10.0.5 software (SPSS Benelux BV, Gorinchem, The Netherlands). Differences in levels of *CA9* expression in samples from patients categorised by clinicopathological characteristics, used as grouping variables, were assessed with nonparametric Mann–Whitney *U* or Kruskall–Wallis testing where appropriate. RFS time (defined as the time from surgery until diagnosis of recurrent disease) was used as main follow-up end point. As adjuvant treatment modality was considered as variable in multivariate analyses, OS time (defined as the time between date of surgery and death by any cause) was not considered as end point, as in some patients OS will be influenced by additional interventions after disease recurrence. The Cox proportional hazards model was used to assess the prognostic value of *CA9* expression in addition to other clinicopathological factors. *CA9* levels were entered as a log-transformed continuous variable. An initial analysis is carried out including all classical clinicopathological factors, with stepwise removal of variables according to their likelihood ratio, to establish a base model. To test for treatment interactions, interaction variables were entered in the model in a second round, including *CA9*, the different treatment modalities (radiotherapy, endocrine therapy and chemotherapy) and their interaction terms (Altman and Lyman, 1998; Harbeck *et al*, 2002; Foekens *et al*, 2003). Two-sided *P*-values below 0.05 were considered to be statistically significant. Cases with >84 months of follow-up were censored at 84 months, because of the rapidly declining number of patients thereafter. This is because after a certain period of observation, patients are frequently redirected to their general practitioner for check-ups and mammography and cease to belong to the outpatients collective of our breast cancer clinic.

## RESULTS

### Immunohistochemistry

**Figure 1:**
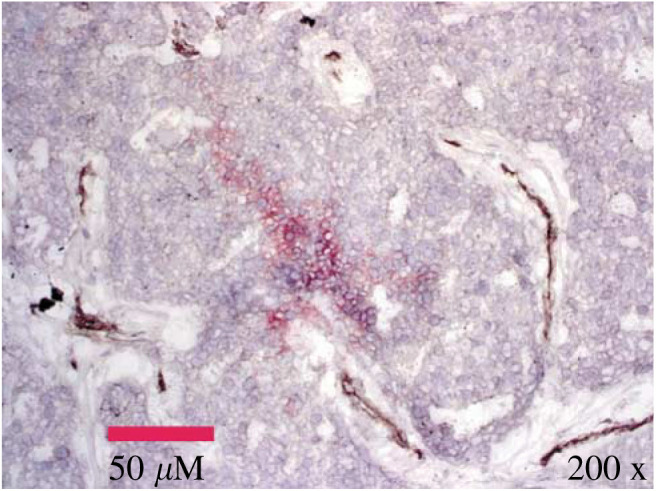
Photomicrograph at X200 magnification illustrating the spatial relationship between CA IX (anti-human G250, red) and tumour vasculature (anti-CD34 staining, brown). Nuclei appear as magenta blue (haematoxylin).

Frozen breast tumour specimens were stained for CA IX. The example shown in Figure 1

shows the typical relationship between membranous CA IX (anti-human G250, red) and surrounding tumour vasculature (anti-CD34 staining, brown). It could be seen that CA IX staining intensity increased at increasing distance from vessels, suggesting an association of CA IX with diffusion limited hypoxia.

### RT–PCR

**Figure 2:**
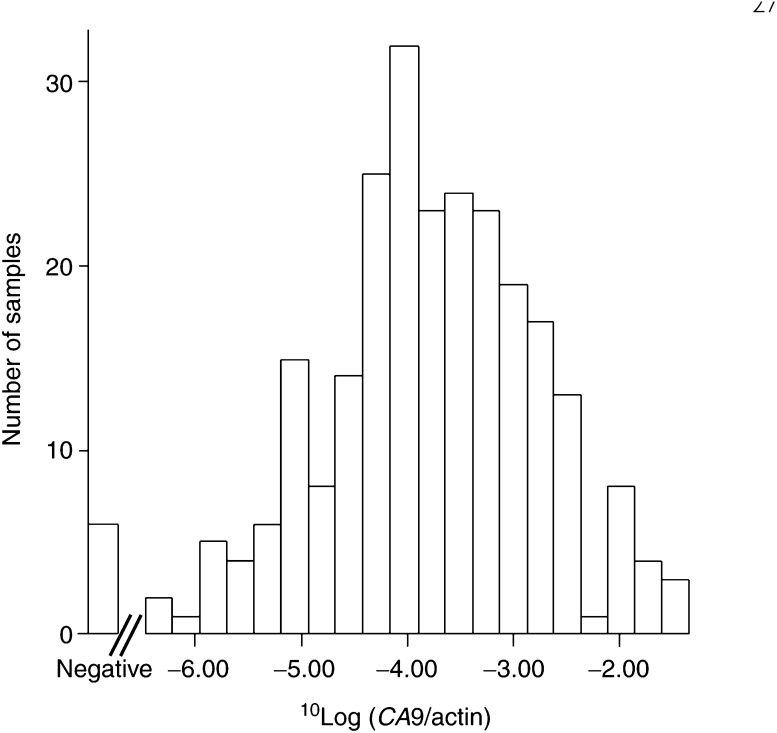
Distribution of *CA9* levels in human breast cancer: six tumour samples were found to be *CA9* negative. The other 247 samples exhibited a basically log-normal distribution of *CA9* levels.

In six tumour samples, *CA9* RT–PCR was negative after 40 rounds of amplification. In the other 247 samples, levels ranged from 3.4*10^−7^ to 4.6*10^−2^ in a log-normal distribution (see Figure 2

). *CA9* levels did not differ statistically significant with age, nodal status, menopausal status, tumour size, type of surgery, radiotherapy or adjuvant systemic treatment (Table 1

**Table 1:** Categorical distributions of baseline characteristics of patients and *CA9*/*β*-actin levels

	**Total group of patients^a^**			
**Variable**	***n***	**% of 253**	**10^−4^ *CA9*/β-actin median (interquartile range)**	**P**
Age (years)				
<50	57	22.5	1.3 (8.8)	
⩾50	196	77.5	1.6 (6.8)	0.656^c^
Nodal status^b^				
Negative	103	40.7	1.0 (7.2)	
1–3 nodes	78	30.8	1.6 (6.2)	
⩾4 nodes	44	17.4	1.3 (7.9)	0.624^d^
Menopausal status				
Premenopausal	64	25.3	1.3 (8.7)	
Postmenopausal	189	74.7	1.6 (6.7)	0.878^c^
Tumour size				
pT1	66	26.1	1.2 (7.0)	
pT2	137	54.2	1.5 (5.8)	
pT3+pT4	46	18.2	3.0 (1.6)	0.611^d^
Histological grade				
I/II	84	33.2	1.1 (2.7)	
III	86	34.0	4.2 (18)	
Unknown	83	32.8	1.8 (6.6)	0.009^d^
ER (fmol mg^−1^ protein)				
<10	88	34.8	7.2 (27)	
⩾10	161	63.6	0.82 (2.7)	<0.001^c^
PgR (fmol mg^−1^ protein)				
<10	103	40.7	6.2 (23)	
⩾10	147	58.1	0.84 (3.1)	<0.001^c^
Surgery				
Mastectomy	183	72.3	1.5 (6.9)	
Breast-saving procedure	70	27.7	1.2 (7.1)	0.902^c^
Adjuvant radiotherapy				
None	68	26.9	1.3 (6.6)	
Any	184	72.7	1.5 (7.4)	0.822^c^
Adjuvant systemic therapy				
None	126	49.8	1.7 (8.2)	
Endocrine	85	33.6	1.5 (5.3)	
Chemo	28	11.1	1.0 (5.6)	
Both	14	5.5	2.8 (15)	0.735^d^

a Owing to missing values data do not always add up to 253.

b All patients with uncertain number of involved lymph nodes are node positive.

c *P* for Mann–Whitney *U* test.

d *P* for Kruskal–Wallis test.

). *CA9* expression levels were found to differ, however, with histological grading (higher in grade III compared to grades I/II, *P*=0.009) and, most notably, with steroid hormone receptor status (higher in steroid receptor negative samples, *P*<0.001 for both ER and PgR, see Table 1).

### Univariate survival analysis

In univariate Cox regression analysis, younger age (*P*=0.018), higher number of involved lymph nodes (*P*<0.001), greater tumour size (*P*=0.011) and higher grade (*P*=0.018) were associated with a poorer RFS (Table 2

**Table 2:** Univariate Cox regression analysis of prognostic value of clinicopathological factors and *CA9* expression for RFS

**Variable**	**HR (95% CI)^a^**	**P**
Age (years)		
<50	1.0	
⩾50	0.56 (0.35–0.91)	0.018
Nodal status		
Negative	1.0	
1–3 nodes	1.97 (1.1–3.5)	
⩾4 nodes	4.23 (2.3–7.7)	<0.001
Menopausal status		
Premenopausal		
Postmenopausal		0.091
Tumour size		
pT1	1.0	
pT2	1.95 (1.1–3.6)	
pT3+pT4	2.90 (1.5–5.8)	0.011
Histological grade		
I/II	1.0	
III	2.24 (1.3–3.9)	
Unknown	1.59 (0.8–2.9)	0.018
ER (fmol mg^−1^ protein)		
<10		
⩾10		0.297
PgR (fmol mg^−1^ protein)		
<10		
⩾10		0.485
Surgery		
Mastectomy		
Breast-saving procedure		0.051
Adjuvant radiotherapy		
None		
Any		0.114
Adjuvant systemic therapy		
None		
Endocrine		
Chemo		
Both		0.680
*CA9* expression continuous	0.99 (0.80–1.22)	0.926

a Hazard ratio (95% CI).

). In contrast to these factors, *C49* levels were not significantly associated with RFS (*P*=0.926, hazard ratio (HR)=0.99, 95% CI=0.80–1.22) in the total cohort of 253 patients for RFS.

### Multivariate survival analysis

In multivariate analysis (Table 3

**Table 3:** Multivariate^a^ Cox regression analysis

**Variable**	**HR (95% CI)^b^**	**P**
*Base model*		
Age (years)		0.146
⩾50 *vs* <50	0.61 (0.31–1.19)	
Tumour size		0.140
pT2 *vs* pT1	1.95 (0.98–3.86)	
pT3+4 *vs* pT1	1.96 (0.90–4.28)	
Histological grade		0.019
III *vs* I/II	2.20 (1.21–4.03)	
No. of involved lymph nodes		<0.001
1–3 *vs* node negative	6.27 (2.89–13.6)	
⩾4 *vs* node negative	13.1 (6.12–27.9)	
ER status^c^		0.852
Positive *vs* negative	1.08 (0.49–2.39)	
PgR status^c^		0.858
Positive *vs* negative	0.96 (0.58–1.58)	
		
*Additions to model*		
*CA9*		0.018
Log-transformed	0.77 (0.62–0.96)	
Adjuvant radiotherapy		0.128
Yes *vs* no	0.60 (0.31–1.16)	
Adjuvant chemotherapy		0.828
Yes *vs* no	0.76 (0.06–9.25)	
Adjuvant endocrine therapy		0.889
Yes *vs* no	1.15 (0.17–7.96)	
Interaction, radiotherapy × *CA9*		0.624
Yes and log-transformed *vs* no and log-transformed	0.89 (0.54–1.44)	
Interaction, chemotherapy × *CA9*		0.009
Yes and log-transformed *vs* no and log-transformed	1.31 (1.07–1.61)	
Interaction, endocrine therapy × *CA9*		<0.001
Yes and log-transformed *vs* no and log-transformed	1.41 (1.20–1.66)	

The analysis was conducted in two blocks. Firstly, a model including all established clinicopathological factors was fitted. In a second block, the contributions of*CA9* (as a continuous, log-transformed variable), treatment and interactions were investigated.

a The final multivariate model included 220 patients.

b Hazard ratio (95% CI) of multivariate analysis.

c Cut-off point used for ER and PgR, 10 fmol mg^−1^ protein.

), histological grade (*P*=0.019) and number of involved lymph nodes (*P*<0.001) remained as factors contributing significantly to RFS. Subsequently, *CA9*, treatment modalities and their interactions were entered. *CA9* (*P*=0.018, HR=0.77, 95% CI=0.62–0.96), the interaction of chemotherapy with *CA9* (*P*=0.009, HR=1.31, 95% CI=1.07–1.61) and the interaction of endocrine therapy with *CA9* (*P*<0.001, HR=1.41, 95% CI=1.20–1.66) all contributed significantly to the final model. These results indicate that *CA9* levels can be used to discriminate patients who are more likely to be resistant to endocrine and/or chemotherapy.

## DISCUSSION

Here, we are the first to show that *CA9* expression as measured by real-time RT–PCR is related to poor outcome after adjuvant treatment in unilateral, invasive breast cancer. Furthermore, *CA9* expression is mainly found in high-grade, steroid receptor negative cancer tissues.

Our approach, that is, the quantification of mRNA by real-time fluorescence RT–PCR, has several advantages over protein detection by immunohistochemistry (IHC). It has the potential of quantification over a wide range of levels and is extremely sensitive. It can be applied to small tissue samples, even after processing the tissues by powdering using a dismembrator for steroid hormone receptor assays as we show here. Finally, antibody-based methods can be less specific, and can be differentially hampered by post-translational modifications, making a proper comparison of data between institutions difficult. On the other hand, IHC methods can be more easily entered into routine clinical practice, and give additional information on the (sub)cellular and tissular expression of CA IX.

A total of 40–50% of the patients with node-positive breast cancer will develop distant metastases within 5 years after primary surgery despite the treatment with adjuvant systemic therapy (EBCTCG, 1996 1998). Thus, identifying patients who will benefit from the other, or more intense treatment protocols is clinically important. CA IX has been related to prognosis and prediction of radio- and/or chemotherapy success in several types of solid tumours (Giatromanolaki *et al*, 2001; Koukourakis *et al*, 2001; Loncaster *et al*, 2001; Hui *et al*, 2002; Turner *et al*, 2002). Its association with prognosis has been reported earlier in breast cancer (Chia *et al*, 2001), although this study included patients treated by adjuvant systemic therapy. The true prognostic value, however, of a given factor should be assessed in patients in the absence of adjuvant treatment (Hayes *et al*, 1998; Henderson and Patek, 1998; Barratt *et al*, 2002; Mori *et al*, 2002). Predictive factors can be used to predict response or lack of response to a particular therapy (Mori *et al*, 2002). Each therapy should be evaluated independently in patient cohorts defined by a predictive factor (Henderson and Patek, 1998). This can be best done in a prospective randomised clinical trial, which is not always feasible. On the other hand, large retrospective data sets containing substantial patient numbers with and without therapy are available for analysis. It is very difficult to draw conclusions on the predictive value of a marker because treatment decisions with regard to adjuvant systemic therapy were based primarily on consensus recommendations at that time, leading to selection bias etc. However, using multivariate analysis with interaction variables is one way to establish the predictive value of a factor from retrospective studies (Altman and Lyman, 1998; Harbeck *et al*, 2002; Foekens *et al*, 2003). In the current study, *CA9* is shown to be an independent predictor of RFS after adjuvant treatment in invasive resectable breast cancer patients, also when corrected for other clinicopathological factors. Indeed, in multivariate Cox regression analysis, a highly significant interaction between endocrine and chemotherapy treatment and *CA9* expression could be established.

Hypoxia is thought to be involved in the development of a radio- or chemotherapy-resistant phenotype of solid tumours. The evidence so far on an association of hypoxia, or factors such as *CA9* that are induced by hypoxia, with resistance to endocrine therapy, however, is scarce. Hypoxia has been shown to lead to hormone insensitivity by ER degradation in breast cancer cell lines (Kurebayashi *et al*, 2001; Stoner *et al*, 2002). The level of VEGF, which is also induced under hypoxic conditions (Wykoff *et al*, 2000; Lal *et al*, 2001), predicts relapse in patients with breast cancer receiving adjuvant endocrine therapy more significantly than after polychemotherapy (Gasparini *et al*, 1999; Linderholm *et al*, 2000). Here, we show that hypoxia-associated *CA9* expression is highest in high-grade, ER-negative breast tumours, which is in line with some (Chia *et al*, 2001), but not all (Bartosova *et al*, 2002), earlier studies. Interestingly, the latter study on *CA9* in breast cancer (Bartosova *et al*, 2002) reported a, albeit weak, correlation with Her2/*neu*, which is known to be associated with the acquirement of hormone insensitivity in breast tumours. Thus, either the reversed correlation between *CA9* and ER status, or its positive correlation with Her2/*neu*, could explain the endocrine therapy resistance in tumours expressing high levels of *CA9*.

In conclusion, we describe here that *CA9* is detectable in breast tumours and is associated with resistance to both adjuvant chemotherapy and endocrine therapy. Thus, *CA9* could aid in the selection of patients who will not benefit from adjuvant therapy and whose prognosis will more likely improve with other treatment modalities.

## References

[bib1] Altman DG, Lyman GH (1998) Methodological challenges in the evaluation of prognostic factors in breast cancer. Breast Cancer Res Treat 52: 289–303 10066088 10.1023/a:1006193704132

[bib2] Barratt PL, Seymour MT, Stenning SP, Georgiades I, Walker C, Birbeck K, Quirke P, UKCCCR AXIS trial collaborators (2002) DNA markers predicting benefit from adjuvant fluorouracil in patients with colon cancer: a molecular study. Lancet 360: 1381–1391, doi:10.1016/S0140-6736(02)11402-4 12423985 10.1016/s0140-6736(02)11402-4

[bib3] Bartosova M, Parkkila S, Pohlodek K, Karttunen TJ, Galbavy S, Mucha V, Harris AL, Pastorek J, Pastorekova S (2002) Expression of carbonic anhydrase IX in breast is associated with malignant tissues and is related to overexpression of c-erbB2. J Pathol 197: 314–321, doi:10.1002/path.1120 12115877 10.1002/path.1120

[bib4] Chia SK, Wykoff CC, Watson PH, Han C, Leek RD, Pastorek J, Gatter KC, Ratcliffe P, Harris AL (2001) Prognostic significance of a novel hypoxia-regulated marker, carbonic anhydrase IX, in invasive breast carcinoma. J Clin Oncol 19: 3660–3668 11504747 10.1200/JCO.2001.19.16.3660

[bib5] Early Breast Cancer Trialists' Collaborative Group (EBCTCG) (1996) Tamoxifen for early breast cancer: an overview of the randomised trials. Lancet 351: 1451–1467, doi:10.1016/S0140-6736(97)11423-4 9605801

[bib6] Early Breast Cancer Trialists' Collaborative Group (EBCTCG) (1998) Poly-chemotherapy for early breast cancer: an overview of the randomised trials. Lancet 352: 930–942, doi:10.1016/S0140-6736(98)03301-7 9752815

[bib8] Foekens JA, Ries C, Look MP, Gippner-Steppert C, Klijn JG, Jochum M (2003) The prognostic value of polymorphonuclear leukocyte elastase in patients with primary breast cancer. Cancer Res 63: 337–341 12543785

[bib9] Gasparini G, Toi M, Miceli R, Vermeulen PB, Dittadi R, Biganzoli E, Morabito A, Fanelli M, Gatti C, Suzuki H, Tominaga T, Dirix LY, Gion M (1999) Clinical relevance of vascular endothelial growth factor and thymidine phosphorylase in patients with node-positive breast cancer treated with either adjuvant chemotherapy or hormone therapy. Cancer J Sci Am 5: 101–111 10198732

[bib10] Giatromanolaki A, Koukourakis MI, Sivridis E, Pastorek J, Wykoff CC, Gatter KC, Harris AL (2001) Expression of hypoxia-inducible carbonic anhydrase-9 relates to angiogenic pathways and independently to poor outcome in non-small cell lung cancer. Cancer Res 61: 7992–7998 11691824

[bib11] Harbeck N, Kates RE, Look MP, Meijer-Van Gelder ME, Klijn JG, Kruger A, Kiechle M, Janicke F, Schmitt M, Foekens JA (2002) Enhanced benefit from adjuvant chemotherapy in breast cancer patients classified high-risk according to urokinase-type plasminogen activator (uPA) and plasminogen activator inhibitor type 1 (*n*=3424). Cancer Res 62: 4617–4622 12183417

[bib12] Hayes DF, Trock B, Harris AL (1998) Assessing the clinical impact of prognostic factors: when is “statistically significant” clinically useful? Breast Cancer Res Treat 52: 305–319 10066089 10.1023/a:1006197805041

[bib13] Henderson IC, Patek AJ (1998) The relationship between prognostic and predictive factors in the management of breast cancer. Breast Cancer Res Treat 52: 261–288 10066087 10.1023/a:1006141703224

[bib14] Höckel M, Schlenger K, Aral B, Mitze M, Schaffer U, Vaupel P (1996a) Association between tumor hypoxia and malignant progression in advanced cancer of the uterine cervix. Cancer Res 56: 4509–4515 8813149

[bib15] Höckel M, Schlenger K, Mitze M, Schaffer U, Vaupel P (1996b) Hypoxia and radiation response in human tumors. Semin Radiat Oncol 6: 3–9 10717157 10.1053/SRAO0060003

[bib16] Hui EP, Chan AT, Pezzella F, Turley H, To KF, Poon TC, Zee B, Mo F, Teo PM, Huang DP, Gatter KC, Johnson PJ, Harris AL (2002) Coexpression of hypoxia-inducible factors 1alpha and 2alpha, carbonic anhydrase IX, and vascular endothelial growth factor in nasopharyngeal carcinoma and relationship to survival. Clin Cancer Res 8: 2595–2604 12171890

[bib17] Koukourakis MI, Giatromanolaki A, Sivridis E, Simopoulos K, Pastorek J, Wykoff CC, Gatter KC, Harris AL (2001) Hypoxia-regulated carbonic anhydrase-9 (CA9) relates to poor vascularization and resistance of squamous cell head and neck cancer to chemoradiotherapy. Clin Cancer Res 7: 3399–33403 11705854

[bib18] Kurebayashi J, Otsuki T, Moriya T, Sonoo H (2001) Hypoxia reduces hormone responsiveness of human breast cancer cells. Jpn J Cancer Res 92: 1093–1101 11676860 10.1111/j.1349-7006.2001.tb01064.x PMC5926610

[bib19] Lal A, Peters H, St Croix B, Haroon ZA, Dewhirst MW, Strausberg RL, Kaanders JH, van der Kogel AJ, Riggins GJ (2001) Transcriptional response to hypoxia in human tumors. J Natl Cancer Inst 93: 1337–1343 11535709 10.1093/jnci/93.17.1337

[bib20] Liao SY, Aurelio ON, Jan K, Zavada J, Stanbridge EJ (1997) Identification of the MN/CA9 protein as a reliable diagnostic biomarker of clear cell carcinoma of the kidney. Cancer Res 57: 2827–2831 9230182

[bib21] Linderholm B, Grankvist K, Wilking N, Johansson M, Tavelin B, Henriksson R (2000) Correlation of vascular endothelial growth factor content with recurrences, survival, and first relapse site in primary node-positive breast carcinoma after adjuvant treatment. J Clin Oncol 18: 1423–1431 10735889 10.1200/JCO.2000.18.7.1423

[bib22] Loncaster JA, Harris AL, Davidson SE, Logue JP, Hunter RD, Wycoff CC, Pastorek J, Ratcliffe PJ, Stratford IJ, West CM (2001) Carbonic anhydrase (CA IX) expression, a potential new intrinsic marker of hypoxia: correlations with tumor oxygen measurements and prognosis in locally advanced carcinoma of the cervix. Cancer Res 61: 6394–6399 11522632

[bib23] Luk CK, Veinot-Drebot L, Tjan E, Tannock IF (1990) Effect of transient hypoxia on sensitivity to doxorubicin in human and murine cell lines. J Natl Cancer Inst 82: 684–692 1969493 10.1093/jnci/82.8.684

[bib25] Mori I, Yang Q, Kakudo K (2002) Predictive and prognostic markers for invasive breast cancer. Pathol Int 52: 186–194 11972862 10.1046/j.1440-1827.2002.01335.x

[bib26] Olive PL, Aquino-Parsons C, MacPhail SH, Liao SY, Raleigh JA, Lerman MI, Stanbridge EJ (2001) Carbonic anhydrase 9 as an endogenous marker for hypoxic cells in cervical cancer. Cancer Res 61: 8924–8929 11751418

[bib27] Opavsky R, Pastorekova S, Zelnik V, Gibadulinova A, Stanbridge EJ, Zavada J, Kettmann R, Pastorek J (1996) Human MN/CA9 gene, a novel member of the carbonic anhydrase family: structure and exon to protein domain relationships. Genomics 33: 480–487, doi:10.1006/geno.1996.0223 8661007 10.1006/geno.1996.0223

[bib28] Rofstad EK (2000) Microenvironment-induced cancer metastasis. Int J Radial Biol 76: 589–605 10.1080/095530000138259 10866281

[bib29] Saarnio J, Parkkila S, Parkkila AK, Haukipuro K, Pastorekova S, Pastorek J, Kairaluoma MI, Karttunen TJ (1998) Immunohistochemical study of colorectal tumors for expression of a novel transmembrane carbonic anhydrase, MN/CA IX, with potential value as a marker of cell proliferation. Am J Pathol 153: 279–285 9665489 10.1016/S0002-9440(10)65569-1 PMC1852958

[bib31] Stoner M, Saville B, Wormke M, Dean D, Burghardt R, Safe S (2002) Hypoxia induces proteasome-dependent degradation of estrogen receptor alpha in ZR-75 breast cancer cells. Mol Endocrinol 16: 2231–2242, doi:10.1210/me.2001-0347 12351689 10.1210/me.2001-0347

[bib32] Turner KJ, Crew JP, Wykoff CC, Watson PH, Poulsom R, Pastorek J, Ratcliffe PJ, Cranston D, Harris AL (2002) The hypoxia-inducible genes VEGF and CA9 are differentially regulated in superficial *vs* invasive bladder cancer. Br J Cancer 86: 1276–1282, doi:10.1038/sj/bjc/6600215 11953885 10.1038/sj.bjc.6600215 PMC2375338

[bib33] Vermylen P, Roufosse C, Burny A, Verhest A, Bosschaerts T, Pastorekova S, Ninane V, Sculier JP (1999) Carbonic anhydrase IX antigen differentiates between preneoplastic malignant lesions in non-small cell lung carcinoma. Eur Respir J 14: 806–811 10573225 10.1034/j.1399-3003.1999.14d14.x

[bib34] Wykoff CC, Beasley N, Watson PH, Campo L, Chia SK, English R, Pastorek J, Sly WS, Ratcliffe P, Harris AL (2001) Expression of the hypoxia-inducible and tumor-associated carbonic anhydrases in ductal carcinoma *in situ* of the breast. Am J Pathol 158: 1011–1019 11238049 10.1016/S0002-9440(10)64048-5 PMC1850356

[bib35] Wykoff CC, Beasley NJ, Watson PH, Turner KJ, Pastorek J, Sibtain A, Wilson GD, Turley H, Talks KL, Maxwell PH, Pugh CW, Ratcliffe PJ, Harris AL (2000) Hypoxia-inducible expression of tumor-associated carbonic anhydrases. Cancer Res 60: 7075–7083 11156414

